# Carboplatin plus paclitaxel in extensive small cell lung cancer: a multicentre phase 2 study

**DOI:** 10.1054/bjoc.2000.1541

**Published:** 2001-01-01

**Authors:** C Gridelli, L Manzione, F Perrone, E Veltri, R Cioffi, M Grazia Caprio, L Frontini, A Rossi, E Barletta, M L Barzelloni, D Bilancia, C Gallo

**Affiliations:** Oncologia Medica B, Istituto Nazionale Tumori, Naples; Oncologia Medica, Ospedale S. Carlo, Potenza; Ufficio Sperimentazioni Cliniche Controllate, Istituto Nazionale Tumori, Naples; Oncologia Medica, Ospedale S. Maria Goretti, Latina; Pneumologia Oncologica, Ospedale Civile, Caserta; Oncologia Medica, Az. Ospedaliera Rummo, Benevento; Oncologia Medica, Ospedale S. Paolo, Milan; Statistica Medica, Seconda Università, Naples

**Keywords:** paclitaxel, carboplatin, small cell lung cancer

## Abstract

A multicentre phase 2 trial (single-stage design) was undertaken to test the efficacy and toxicity of carboplatin (AUC 6 according to Calvert) plus paclitaxel (175 mg/m^2^3-h infusion) every 4 weeks in the first line treatment of patients affected by extensive small cell lung cancer. The primary end-point of the trial was the objective response rate. 31 objective responses among 50 patients were considered necessary to proceed to a phase 3 trial. 48 patients were enrolled (median age 59 years). Treatment was very well tolerated. 3 patients (6.2%) had a complete response and 23 (47.9%) a partial response, for an overall response rate of 54.2% (95% CI: 39.2–68.6) Median time to progression was 5.7 months (95% CI: 5.2–6.2). Median survival was 9.6 months (95% CI: 7.2–14.6), with a median follow-up time of alive patients of 12 months. At 1 year, the probability of being progression-free or alive was 0.16 and 0.43, respectively. In conclusion, carboplatin plus paclitaxel as given in the present study is very well tolerated but not sufficiently active to warrant phase 3 comparison with standard chemotherapy regimens. © 2001 Cancer Research Campaign http://www.bjcancer.com


British Journal of Cancer (2001) **84**(1), 38–41
© 2001 Cancer Research Campaign
doi: 10.1054/ bjoc.2000.1541, available online at http://www.idealibrary.com on IDEAL®

http://www.bjcancer.com

### Carboplatin plus paclitaxel in extensive small cell lung cancer: a multicentre phase 2 study

**C Gridelli[1], L Manzione[2], F Perrone[3], E Veltri[4], R Cioffi[5], M Grazia Caprio[6], L Frontini[7], A Rossi[1,2], E Barletta[1], ML Barzelloni[1], D Bilancia[2] and C Gallo[8]**

[1]Oncologia Medica B, Istituto Nazionale Tumori, Naples; [2]Oncologia Medica, Ospedale S. Carlo, Potenza; [3]Ufficio Sperimentazioni Cliniche Controllate, Istituto Nazionale Tumori, Naples; [4]Oncologia Medica, Ospedale S. Maria Goretti, Latina; [5]Pneumologia Oncologica, Ospedale Civile, Caserta; [6]Oncologia Medica, Az. Ospedaliera Rummo, Benevento; [7]Oncologia Medica, Ospedale S. Paolo, Milan; [8]Statistica Medica, Seconda Università, Naples

**Summary** A multicentre phase 2 trial (single-stage design) was undertaken to test the efficacy and toxicity of carboplatin (AUC 6 according to Calvert) plus paclitaxel (175 mg/m$^2$ 3-h infusion) every 4 weeks in the first line treatment of patients affected by extensive small cell lung cancer. The primary end-point of the trial was the objective response rate. 31 objective responses among 50 patients were considered necessary to proceed to a phase 3 trial. 48 patients were enrolled (median age 59 years). Treatment was very well tolerated. 3 patients (6.2%) had a complete response and 23 (47.9%) a partial response, for an overall response rate of 54.2% (95% CI: 39.2–68.6). Median time to progression was 5.7 months (95% CI: 5.2–6.2). Median survival was 9.6 months (95% CI: 7.2–14.6), with a median follow-up time of alive patients of 12 months. At 1 year, the probability of being progression-free or alive was 0.16 and 0.43, respectively. In conclusion, carboplatin plus paclitaxel as given in the present study is very well tolerated but not sufficiently active to warrant phase 3 comparison with standard chemotherapy regimens. © 2001 Cancer Research Campaign http://www.bjcancer.com

**Keywords:** paclitaxel; carboplatin; small cell lung cancer

Small-cell lung cancer (SCLC) has an ominous prognosis despite being susceptible to both chemotherapy and radiotherapy. Median survival of patients affected by extensive disease ranges between 8 and 11 months, with minimal or no chances of long-term survival (Ihde, 1984). Chemotherapy of extensive SCLC results in a response rate of 60–70%; a complete response is obtained in 10–30% of cases. Three chemotherapy regimens are widely used to treat SCLC: CAV (cyclophosphamide, adriamicin, vincristine) (Feld et al, 1984), CDE (cyclophosphamide, doxorubicin, etoposide) (Aisner et al, 1986) and PE (cisplatin, etoposide) (Einhorn, 1986). The three regimens produce similar response rates.

Carboplatin, as single agent, was successfully used to treat SCLC in the mid 1980s (Smith and Evans, 1985; Jacobs et al, 1987). Two randomized trials showed that there was no difference in terms of efficacy between carboplatin-containing and cisplatin-containing polychemotherapy (Kosmidis et al, 1994; Lassen et al, 1996). Thanks also to its lower toxicity, carboplatin has become the most widely used drug in combination with etoposide (Smith et al 1987; Evans et al, 1988; Bishop, 1992).

Various newer drugs are under investigation: topotecan (Schiller et al, 1998), vinorelbine (Gridelli et al, 1998), gemcitabine (Cormier et al, 1994) and paclitaxel. The latter, as a single agent, resulted in a 53% response rate in patients with extensive-stage SCLC (Ettingen et al, 1995). Paclitaxel has been associated with either carboplatin or cisplatin and, in 3-drug regimens, with etoposide (Hainsworth et al, 1997; Glisson et al, 1999). Carboplatin plus paclitaxel has proved successful as second-line treatment (Groen et al, 1999). There are no studies on the first-line use of this combination.

*Received 17 May 2000*
*Revised 15 August 2000*
*Accepted 13 September 2000*

*Correspondence to:* C Gridelli

The aim of our study was to evaluate whether carboplatin plus paclitaxel is sufficiently active in the treatment of adult patients with extensive SCLC to warrant comparative trials with standard regimens.

### PATIENTS AND METHODS

### Study design

We conducted a multicentre phase 2 trial, the main outcome being response rate. A lowest acceptable probability of response ($p_0$) of 0.50 and a desired probability of response ($p_1$) of 0.70 were used to calculate sample size. With a type I error of 0.05 and a type II error of 0.10, at least 31 objective responses out of 50 patients were required to proceed to phase III testing. The trial was approved by the ethics committees of the participating institutions.

### Eligibility criteria

Patients aged 65 years or less were eligible for the trial if they had a cytological or histological diagnosis of extensive SCLC and a performance status less than or equal to 2 on the ECOG scale. Patients previously treated with chemotherapy or radiotherapy or with a history of another type of cancer (with the exception of non-melanomatous skin cancer and in situ radically resected cervix cancer) were excluded. Normal bone marrow, kidney, liver and heart function were prerequisites for trial entry. Patients with asymptomatic brain metastases were not excluded. All patients gave their written informed consent to the study.

Basal evaluation consisted of clinical examination, haematological and biochemical assessment and cardiological evaluation with ECG. Staging procedure consisted of 2-view chest X-ray; brain, chest, abdomen contrast enhanced CT-scans; radionuclidic bone scan with X-ray details of hot spots.

### Drug schedule

Carboplatin was given i.v. to reach a dose of AUC 6, diluted in 250 cc normal saline solution in 30 min on day 1; paclitaxel was given i.v. at a dose of 175 mg/m$^2$, diluted in 500 cc normal saline in a 3-h infusion on day 1. Creatinine clearance was either measured or calculated with the Jelliffe formula (Jelliffe, 1997); the carboplatin dose was calculated according to Calvert's formula (Calvert et al, 1989). Antiemetic medication consisted of 5HT3 antagonists (at a standard dose) on day 1. 30 min before paclitaxel, patients were given 20 mg dexamethasone plus 100 mg ranitidine plus 50 mg prometazine i.v.

Cycles were repeated every 4 weeks for a maximum of 6 times. Dose reductions were not planned. Cycles could be delayed up to 2 weeks in case of neutrophils <1.500 mm c$^{-1}$ or platelets <100 000 mm c$^{-1}$ on the day of planned treatment. Treatment was stopped in cases of a delay of 2 weeks and persisting toxicity. Response was evaluated at the end of the third cycle, and only responding patients remained on treatment. Investigators were free to decide whether or not to administer prophylactic G-CSF.

### Calculation of dose parameters

To verify patients' compliance to the treatment schedule, dose parameters were calculated for paclitaxel alone. The total delivered dose of paclitaxel is the sum of the single delivered doses. Time-on-treatment is the interval between day 1 of the first cycle up to day 28 of the last cycle. Delivered dose-intensity is the ratio between total delivered dose and time-on-treatment, and is expressed as mg/m$^2$ week$^{-1}$. Relative dose-intensity of paclitaxel is the ratio between delivered dose-intensity and planned dose-intensity (43.75 mg/m$^2$ week$^{-1}$).

### Scales for toxicity and response assessment

The WHO graded scale (Miller et al, 1981) was used to score toxicity; scoring of fatigue and headache were revised a posteriori and codified according to the Common Toxicity Criteria of the National Cancer Institute of Canada (version 2.0). Toxicity was assessed before each cycle of chemotherapy. Toxicity was assessed on the worst data for each patient across all cycles of chemotherapy. Objective responses were evaluated according to the WHO scale (Miller et al, 1981) at the end of chemotherapy cycles 3 and 6 by repeating staging procedures. The best response was recorded for each patient. Response evaluation was anticipated in case of clinically evident or suspected disease progression. The objective response rate is the proportion of complete plus partial responses on all patients.

### Statistical analysis

For response rate, we calculated (Geigy Scientific Tables) 95% exact binomial confidence intervals (CI). We used contingency tables to evaluate the association between the patients' baseline characteristics and objective response (coded as binary variable: no/yes), and the chi-square test to test statistical significance, using test for trend for PS and number of neoplastic sites. Fisher's exact test was used to test association of toxicity with G-CSF administration. Progression-free survival was defined as the interval between day 1 of cycle 1 up to progression of disease or death, whichever occurred first. Overall survival was calculated as the interval between day 1 of cycle 1 and date of death or date of the last follow-up visit. Progression-free and overall survival curves were estimated with the Kaplan-Meier product limit method (Kaplan and Meier, 1958); the 95% CI of median values were calculated according to Brookmeier and Crowley (BMDP Statistical Software, Los Angeles, CA, University of California Press, 1992).

**Table 1** Characteristics of patients ($n$ = 48)

| | |
|---|---|
| Sex, No. (%) | |
| Male | 42 (87.5%) |
| Female | 6 (12.5%) |
| Age, years | |
| Median | 59 |
| Range | 36–65 |
| ECOG performance status score, No. (%) | |
| 0 | 7 (14.6) |
| 1 | 32 (66.7) |
| 2 | 9 (18.8) |
| Brain metastases, No. (%) | |
| No | 44 (91.7) |
| Yes (asymptomatic) | 4 (8.3) |
| Previous radiotherapy, No. (%) | |
| No | 48 (100) |
| Neoplastic sites, No. (%) | |
| 1 | 2 (4.2) |
| 2 | 9 (18.8) |
| 3 | 22 (45.8) |
| 4 | 14 (29.2) |
| 5 | 1 (2.1) |

### RESULTS

From March 1997 to October 1999, 48 patients (males: 87.5%) entered the trial (Table 1). Median age was 59 years (range 36–65). Performance status was impaired in most patients (14.6% were in EOCG category 0 and 66.7% in category 1). 4 patients had asymptomatic brain metastases at entry. No patient had received previous chemotherapy or radiotherapy. The median number of neoplastic sites was 3 (range 1–5). 25 (52.1%) patients received all the planned 6 cycles of treatment. Treatment was stopped because of progressive disease in 14 (29.2%) patients, toxicity in 2 (4.2%) and refusal in 7 (14.6%). Prophylactic G-CSF from cycle 1 was given to 17 (35.4%) patients. Another patient required G-CSF because of grade 4 neutropenia at the first cycle; this patient also received prophylactic G-CSF in subsequent cycles.

The median relative delivered dose-intensity (RDDI) of paclitaxel was 0.99 (range 0.83–1.19). There was a deviation from the protocol in 2 patients, i.e., recycling treatment was given every 21 instead of 28 days. There was no difference ($P$ = 0.52) in the median RDDI between patients who did not receive prophylactic G-CSF ($n$ = 30, mean RDDI = 0.98) and those who did ($n$ = 18, mean RDDI = 1.01).

Overall, toxicity was mild (Table 2); only two patients stopped treatment because of toxicity. One patient had grade 4 hepatic toxicity after cycle 4. This patient had grade 1 hepatic toxicity during cycles 2 and 3, but had recovered before the subsequent dose of chemotherapy. Another patient developed allergy after administration of cycle 1.

There was no difference in incidence and degree of toxicity between patients receiving and those not receiving prophylactic

© 2001 Cancer Research Campaign 

**Table 2** Worst grade of toxicity

| Toxicity (grade) | No. (%) of patients |
|---|---|
| Leukopenia (4) | – |
| Neutropenia (4) | 1 (2.1) |
| Thrombocytopenia (2–3) | 3 (6.3) |
| Anaemia (2–3) | 4 (8.3) |
| Infections (3) | 1 (2.1) |
| Vomiting (2–3) | 6 (12.5) |
| Diarrhoea (2) | 1 (2.1) |
| Oral mucositis (2) | 2 (4.2) |
| Fatigue (2–3) | 15 (31.3) |
| Headache (grade 3) | 1 (2.1) |
| Allergy (1–2) | 2 (4.2) |
| Skin (1) | 1 (2.1) |
| Fever (1) | 1 (2.1) |
| Cardiac (1) | 1 (2.1) |
| Respiratory (1–2) | 3 (6.3) |
| Renal (1) | 1 (2.1) |
| Hepatic (4) | 1 (2.1) |
| Constipation (1) | 6 (12.5) |
| Peripheral neurotoxicity (1–2) | 3 (6.3) |
| Central nervous system (1) | 1 (2.1) |
| Hair loss (2–3) | 20 (41.7) |

**Table 3** Objective results

| Outcome | No. (%) | 95% CI |
|---|---|---|
| Objective response | | |
| Complete response | 3 (6.2) | |
| Partial response | 23 (47.9) | |
| Stable disease | 8 (16.7) | |
| Progressive disease | 11 (22.9) | |
| Restaging not performed[a] | 3 (6.2) | |
| Response rate | 26 (54.2) | 39.2–68.6 |
| Progressed patients | 43 (89.6%) | |
| Median time-to-progression (months) | | 5.7 |

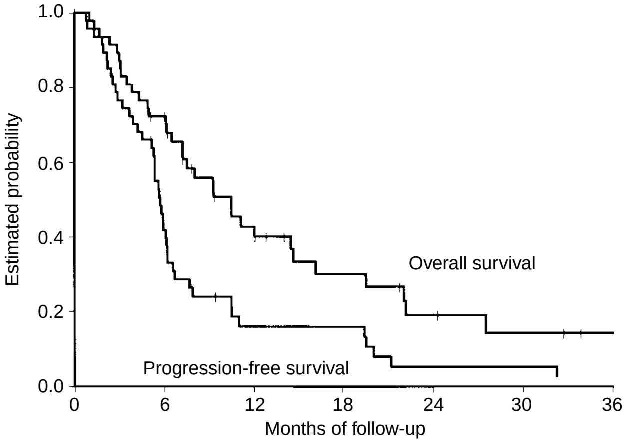

**Figure 1** Kaplan-Meier estimated progression-free and overall survival curves

G-CSF, with the exception of grade 2–3 thrombocytopenia, which occurred only in patients receiving G-CSF ($P = 0.05$).

3 patients (6.2%) had a complete response and 23 (47.9%) a partial response (Table 3), resulting in an overall response rate of 54.2% (95% exact CI: 39.2–68.6). Sex, age and performance status did not affect the probability of response (data not shown). The number of disease sites was correlated ($P = 0.04$) with response: the rate of responder patients decreased from 72.7% to 59.1% and 33.3% in patients with <3, 3 and >3 sites of disease, respectively.

Second-line chemotherapy was given to 13 patients out of 48. 8 of the 13 patients had partially responded to initial chemotherapy, 3 had had a stabilization and 2 had progressed while on treatment. Second-line chemotherapy consisted of combination treatment in 2 cases (ifosfamide + vinorelbine and carboplatin + epirubicin + etoposide, 1 case each) and single agent treatment in 11 cases (topotecan 6 cases, etoposide 3 cases, epirubicin 1 case, vinorelbine 1 case). One partial response was obtained (in the patient receiving the triplet regimen, who had also responded to first-line treatment) and one disease stabilization with topotecan, in a patient who had progressed while on first-line treatment.

As shown in Figure 1, as of March 2000, 43 patients (89.6%) had progressed and 33 (68.7%) had died. Median time-to-progression was 5.7 months (95% CI: 5.2–6.2). Median survival was 9.6 months (95% CI: 7.2–14.6); the median follow-up time of alive patients was 12 months. At 1 year, the probability of being progression-free or alive was, respectively, 0.16 and 0.43.

### DISCUSSION

We found that carboplatin plus paclitaxel is very well tolerated but not sufficiently active to warrant phase 3 comparison with standard chemotherapy regimens for extensive SCLC.

A possible explanation for this negative result is that the dose-intensity of both drugs in our study was lower compared with schedules commonly used in such solid tumours as non-SCLC and ovarian cancer. The low dose-intensity is primarily due to recycling every 4 weeks. In addition, the paclitaxel dose per cycle is in the lower range of doses administered as 3-h infusion in the treatment of other solid tumours.

We elected to test a non-aggressive scheme so as not to deteriorate the patients' quality of life. At present, patients with extensive SCLC have no chance of being cured, little chance of prolonged survival and urgent need of palliation with treatment well balanced in terms of efficacy and toxicity. To our knowledge there are no publications about carboplatin plus paclitaxel as first-line treatment for extensive-stage SCLC patients. However, our results could be expected from two abstracts presented at the 1999 Congress of the American Society of Clinical Oncology. Thomas and colleagues (1999) studied a regimen theoretically more intensive than ours (AUC 6 carboplatin and 200 mg/m$^2$ paclitaxel as 3-h infusion given every 3 weeks); data reported for 35/46 enrolled patients showed a response rate of 67%, 10% complete responses and a median survival of 6 months. Deppermann et al (1999) using the same schedule as Thomas et al obtained a 61% response rate in 75 patients, with 7% complete responses and a median survival of 12 months. Toxicity was important in the latter study: grade 3–4 neutropenia in 43% of cycles and grade 2–3 peripheral neurotoxicity in 29% of cycles. Our results are consistent with these two abstracts, except toxicity was less frequent and severe in our study.

Our data differ from those reported by Groen et al (1999) who studied the same combination (AUC 7 carboplatin plus 175 mg/m$^2$ paclitaxel as 3-h infusion every 3 weeks) as second-line treatment in 34 SCLC patients, most of whom had responded to a previous CDE regimen. Groen and colleagues obtained an impressive 73.5% response rate (with 6% complete responses) and a median survival

© 2001 Cancer Research Campaign exceeding 7 months. The selection of patients with a positive prognostic factor (sensitivity to previous chemotherapy) and other unknown selection biases might explain this divergence. However, ours being a phase 2 study design, we cannot exclude a false-negative result. Nevertheless, our data together with earlier studies (Deppermann, 1999; Thomas, 1999) indicate that the association of paclitaxel with platinum compounds in 2-drug combinations is not a good candidate for phase 3 trials.

A promising approach might be to add paclitaxel to standard drugs, in 3-drug combinations, e.g. carboplatin or cisplatin plus etoposide plus paclitaxel. Kelly et al (1999), in a phase 1 trial of paclitaxel plus etoposide plus cisplatin in extensive disease found that the recommended doses were 175 mg/m$^2$ as 3-h infusion on day 1 for paclitaxel, 80 mg/m$^2$ i.v. on day 1 and 160 mg/m$^2$ p.o. on days 2–3 for etoposide, and 80 mg/m$^2$ i.v. cisplatin on day 1, all recycled every 3 weeks, with support of haemopoietic growth factors. Out of 28 patients treated at 4 dose levels, an 83% response rate was recorded with 10 months median survival. Glisson et al (1999), in a phase 1–2 study of cisplatin plus etoposide plus paclitaxel, which included 41 patients with extensive SCLC, reported the maximum tolerated dose of the 3 drugs to be 75 mg/m$^2$ day 1, 80 mg/m$^2$ days 1–3, 135 mg/m$^2$ in 3 h day 1, respectively, all given i.v. every 3 weeks. Responses were recorded in 90% of patients, and were complete in 16%. In another phase 2 trial of 117 SCLC patients, carboplatin plus oral etoposide plus paclitaxel resulted in an impressive response rate of 98% with complete responses in 71% and median survival not reached at 16 months of follow-up in patients with non-extensive disease. In cases of extensive disease the response rate was 84%, complete responses 21% and 10 months median survival. Overall, as expected, the treatment was very toxic with grade 3–4 neutropenia in 71% of patients, grade 3–4 thrombocytopenia in 24% and grade 3–4 anaemia requiring transfusion in 35%. These data strongly suggest that the 3-drug combination needs to be studied in phase 3 comparisons that explore the impact of toxicity on the quality of life of patients in view of the small survival advantages.

In conclusion, the 2-drug combination with carboplatin plus paclitaxel should undergo further explorative clinical trials before it can be recommended for phase 3 testing.

### ACKNOWLEDGEMENTS

The following institutions participated in the study with one patient enrolled:
Medicina Interna III, Università di Napoli Federico II, Naples
Cattedra di Oncologia, Università, Cagliari
Oncologia Pneumologica V, Ospedale Monaldi, Naples
Oncologia Medica, Ospedale Civile, Alba
Day-Hospital di Oncologia, Ospedale Civile, Polla (SA)
Unità Operativa di Oncologia Medica Sperimentale, IRCCS Oncologico Mater Dei, Bari
We thank Federika Crudele, Giuliana Canzanella and Assunta Caiazzo for data management and secretarial services; and Jean Ann Gilder for editing the text.

### REFERENCES

Aisner J, Whitacre M and Abrams J (1986) Doxorubicin, cyclophosphamide, etoposide and platinum, doxorubicin, cyclophopshamide and etoposide for small cell lung carcinoma of the lung. *J Clin Oncol* **3** (suppl 3): 54–62

Bishop JF (1992) Carboplatin/etoposide in small cell lung cancer. *Oncology* **49**(suppl 1): 11–18

Calvert AH, Newell DR, Gumbrell LA, O'Reilly S, Burnell M, Boxall FE, Siddik ZH, Judson IR, Gore ME and Wiltshaw E (1989) Carboplatin dosage: prospective evaluation of a simple formula based on renal function. *J Clin Oncol* **7**: 1748–1756

Cormier Y, Eisenhauer E, Mudal A, Gregg R, Ayoub J, Goss G, Stewart D, Tarasoff P and Worg D (1994) Gemcitabine is an active new agent in previously untreated extensive small cell lung cancer (SCLC). *Ann Oncol* **5**: 283–285

Deppermann KM, Serke M, Oehm C, Kurzeja A, Riedel U, Lichey HJ, Loddenkemper R, Klockner T and Kettner H (1999) Paclitaxel (TAX) and carboplatin (CBDCA) in advanced SCLC: a phase II study. *Proc Am Soc Clin Oncol* **18**: 482a

Einhorn LH (1986) Initial therapy with cisplatin plus VP-16 in small cell lung cancer. *Semin Oncol* **13**(suppl): 5–9

Ettinger DS, Finkelstein DM, Sarma RP and Johnson DH (1995) Phase II study of paclitaxel in patients with extensive disease small cell lung cancer: an Eastern Cooperative Oncology Group Study. *J Clin Oncol* **13**: 1430–1435

Evans WK, Eisenhauer E, Hughes P, Maroun JA, Ayoub J, Shepherd FA and Feld R (1988) VP-16 and carboplatin in previously untreated patients with extensive small cell lung cancer: a study of the National Cancer Institute of Canada Clinical Trials Group. *Br J Cancer* **58**: 464–468

Feld R, Evans WK and De Boer (1984) Combined modality induction therapy without maintenance chemotherapy for small cell carcinoma of the lung. *J Clin Oncol* **2**: 294–304

Glisson BS, Kurie JM, Perez-Soler R, Fox NJ, Murphy WK, Fossella FV, Lee JS, Ross MB, Nyberg DA, Pisters KMW, Shin DM and Hong WK (1999) Cisplatin, etoposide, and paclitaxel in the treatment of patients with extensive small cell lung carcinoma. *J Clin Oncol* **17**: 2309–2315

Gridelli C, Perrone F, Ianniello GP, Brancaccio L, Iaffaioli RV, Curcio C, D'Aprile M, Cioffi R, Cigolari S, Rossi A, Palazzolo G, Veltri E, Pergola M, De Placido S, Gallo C, Monfardini S and Bianco AR (1998) Carboplatin plus vinorelbine, a new well tolerated and active regimen for the treatment of extensive stage small cell lung cancer: a phase II study. *J Clin Oncol* **16**: 1414–1419

Groen HJM, Fokkem E, Biesma B, Kwa B, van Putten JWG, Postmus PE and Smit EF (1999) Paclitaxel and carboplatin in the treatment of small cell lung cancer patients resistant to cyclophosphamide, doxorubicin, and etoposide: a non cross resistant schedule. *J Clin Oncol* **17**: 927–932

Hainsworth JD, Gray JR, Stroup SL, Kalman LA, Patten JE, Hopkins LG, Thomas M and Greco FA (1997) Paclitaxel, carboplatin, and extended-schedule etoposide in the treatment of small cell lung cancer: comparison of sequential phase II trials using different dose-intensities. *J Clin Oncol* **15**: 3464–3470

Ihde DC (1984) Current status of therapy of small cell carcinoma of the lung. *Cancer* **2**: 2722–2728

Jacobs RH, Bitran J, Deutsch M, Hoffman PC, Sinkule J, Purl S and Golomb HM (1987) Phase II study of carboplatin in previously untreated patients with metastatic small cell lung carcinoma. *Cancer Treat Rep* **71**: 311–312

Jelliffe RW (1973) Creatinine clearance: bedside estimate. *Ann Intern Med* **79**: 604–605

Kaplan EL and Meier P (1958) Nonparametric estimation from incomplete observation. *J Am Stat Assoc* **53**: 457–481

Kelly K, Pan Z, Wood ME, Murphy J and Bunn PA Jr (1999) A phase I study of paclitaxel, etoposide, and cisplatin in extensive stage small cell lung cancer. *Clin Cancer Res* **5**: 3419–3424

Kosmidis PA, Samantas E, Fountzilas G, Paulidis, Apostolopoulou F and Skarlos D (1994) Cisplatin/etoposide versus carboplatin/etoposide chemotherapy and irradiation in small cell lung cancer: a randomized phase III study. *Semin Oncol* **21**(suppl 6): 23–30

Lassen U, Kristjansen PEG, Osterlind K, Bargman B, Sisgaard TC, Hirsch FR, Hansen M, Dombernowsky P and Hansen HH (1996) Superiority of cisplatin or carboplatin in combination with teniposide and vincristine in the induction chemotherapy small cell lung cancer. A randomized trial with 5 years follow-up. *Ann Oncol* **7**: 365–371

Miller AB, Hoogstraten B, Staquet M and Winkler A (1981) Reporting results of cancer treatment. *Cancer* **47**: 207–214

Schiller JH, Kim K, Hutson P, De Vore R, Glick J, Stewart J and Johnson D (1998) Phase II study of topotecan in patients with extensive stage small cell carcinoma of the lung: an Eastern Cooperative Oncology Group Trial. *J Clin Oncol* **14**: 2345–2352

Smith IE and Evans BD (1985) Carboplatin (JM8) as single agent and combination in the treatment of small cell lung cancer. *Cancer Treat Rev* **12**: 73–75

Smith IE, Evans BD, Gore ME, Vincent MD, Repetto L, Yarnold JR and Ford HT (1987) Carboplatin (Paraplatin; JM8) and etoposide (VP-16) as first-line combination therapy for small cell lung cancer. *J Clin Oncol* **5**: 185–189

Thomas P, Lena M, Robinet, Paillotin D, Delaval, Clavier, Bolmes P, Blanchon F, Perdu D, Poirier R, Pommier de Santi P and Kleisbauer JP (1999) Preliminary report on paclitaxel/carboplatin phase II multicentric trial in patients with metastatic small cell lung cancer (SCLC). *Proc Am Soc Clin Oncol* **18**: 519a

© 2001 Cancer Research Campaign 
